# Supplementary material for: Impact of the COVID-19 pandemic on the routine immunization system in Bangladesh
Source: PLoS One. 2025 Oct 14;20(10):e0334503. doi: 10.1371/journal.pone.0334503 (PMC12520360; doi:10.1371/journal.pone.0334503)
Supplement: Attachment — Submitted filename: Author_Response_auresp_2.docx [file pone.0334503.s002.docx]

Response to Academic Editor and Reviewers

Manuscript ID: PONE-D-25-30097R1
Title: *Impact of the COVID-19 pandemic on the routine immunization system in Bangladesh*

Dear Dr. Nomhwange,

We sincerely thank you and the reviewers for your constructive feedback and careful consideration of our manuscript. We are pleased that both reviewers confirmed all prior comments have been addressed and that they found the manuscript technically sound. Below, we provide a point-by-point response to the additional comments from the Academic Editor. All revisions have been incorporated into the revised manuscript.

Academic Editor Comments

1. Please include any study limitations as a subsection before the conclusion.
*Response:* We have added a new subsection titled “Study Limitations” before the conclusion.

2. I suggest a revision of the conclusion section to make it less wordy and more succinct.
*Response:* The conclusion has been carefully revised to present the main findings and implications in a more concise manner.

3. Please also review the current draft for consistency in the referencing style. In some cases, the reference is included at the end of sentences while in other cases this is included within a sentence. I suggest all references are consistently inserted at the end of a statement.
*Response:* We have thoroughly reviewed and edited the reference style to ensure consistency, placing all references at the end of the relevant statement as suggested.

4. You may wish to also include a footnote on the charts/Tables explaining why coverage levels reports are above 100%.
*Response:* We have added a standardized footnote to all relevant tables and figures. The footnote clarifies that coverage levels above 100% result from denominator estimation issues in aggregated reporting systems, which may lead to overestimation. (See Table 1)

We hope these revisions satisfactorily address all comments and improve the manuscript. We greatly appreciate the opportunity to revise and resubmit, and we look forward to your favorable consideration.

With kind regards,
Md. Foyjul Islam (on behalf of all co-authors)
